# Data on the number of passengers using buses in Abashiri city, Hokkaido, from 2013 to 2018

**DOI:** 10.1016/j.dib.2019.104512

**Published:** 2019-09-13

**Authors:** Tomohisa Ueda, Akihisa Akazawa, Yoshimasa Sagane

**Affiliations:** a Department of Business, Natural Resource and Economic Development, Tokyo University of Agriculture, 196 Yasaka, Abashiri, Hokkaido, 099-2493, Japan; b Commerce, Industry, and Labor Deviation, Department of Commerce and Tourism, Abashiri City Office, Higashi 4, Minami 6, Abashiri, 093-8555, Japan; c Department of Food, Aroma, and Cosmetic Chemistry, Tokyo University of Agriculture, 196 Yasaka, Abashiri, Hokkaido, 099-2493, Japan

**Keywords:** Public transportation, Bus, City planning, Abashiri city, Japan

## Abstract

This article relates to data concerning the number of passengers using buses operating in Abashiri city, Hokkaido, Japan, from 2013 to 2018. Data on the number of passengers for three lines—the *shopping* line, the *Tokyo NODAI* line, and the *Hagoromo-danchi* line—were collected by counting the passengers getting on and off the buses at each stop. The survey was conducted for one week each in February, June, and September of each year of the study period. The data showing the number of customers getting on, getting off, or passing each bus stop are provided in Microsoft Excel Worksheets.

**Table:** Specifications Table

Subject area	*Social science*
More specific subject area	*Public transport*
Type of data	*Microsoft Excel Worksheet*
How data was acquired	*Field survey*
Data format	*Raw*
Experimental factors	*The numbers of the passengers getting on and off at or passing by each bus stop.*
Experimental features	*Investigators boarded the bus during peak time and counted the number of passengers. Bus drivers also counted passengers during non-peak times.*
Data source location	*Abashiri city, Hokkaido, Japan.*
Data accessibility	*The data are included in this article.*

**Table:** 

**Value of the Data** • The data presented could assist researchers involved in city planning to understand transportation trends of residents in a city in rural Hokkaido, Japan. • It could assist public transportation researchers addressing the problem concerning the decrease in the use of buses as public transportation by analyzing the data in this article and the population distributions in the city. • In Japan, the number of bus passengers is decreasing despite the necessity of public transport as a means of moving around for the elderly [1]. The data can be very valuable for planning different means of public transportation that may replace buses in the future.

## 1 Data

The data provided (supplementary material 1 to 3) are in the format of Microsoft Excel worksheets and list the numbers of passengers getting on and getting off or passing by at each bus stop for one week of each month mentioned and as indicated by the tabs in the worksheet. Each spreadsheet contains the data for three bus routes operating in Abashiri city: the *Hagoromo-danchi* line (supplementary material 1), the *shopping* line (supplementary material 2), and the *Tokyo NODAI* line (supplementary material 3). The first column of each table in the worksheet lists the names of bus stops on different routes while the columns between the first and last columns contain the dates and numbers of passengers. The last column in each table contains the sum of passengers in each week. The X symbols in the cell mean the bus skipped that particular stop. Each table represents the bus leaving from the first bus stop at the indicated time at the top of the table.

## 2 Experimental design, materials, and methods

### 2.1 Design

Three distinctive routes were chosen for this data: the *shopping* line, which is the route connecting certain shopping malls to several residential areas in the city; the *Tokyo NODAI* line, which is the route that connects the Tokyo University of Agriculture campus in the city to residential areas; and the *Hagoromo-danchi* line, which is the route that connects three hospitals in the city to residential areas. The objective of this survey was to assess the suitability of the routes and schedules of bus transportation services in a city of rural Hokkaido, Japan.

### 2.2 Survey

The surveys were carried out 15 times on the following dates: 2013 (March 10–16, June 21–26, and September 7–13), 2014 (February 22–28, June 23–29), 2015 (March 7–13, June 23–29, and September 8–14), 2016 (February 23–29, June 23–29, and September 9–15), 2017 (February 21–27, June 19–25, and September 9–15), and 2018 (February 20–26). During peak time, an investigator boarded the bus and counted the number of passengers who got on and off the bus at each stop. During non-peak hours, the driver counted and recorded the number of passengers. At each bus stop, the passengers who did not get off the bus were counted as a “pass.”

### 2.3 Aggregation of passenger data

The numbers of passengers for every action were compiled in a worksheet on Microsoft Excel for Mac software (version 16.26; Redmond, WA, USA). The sum of passengers on each day and week were calculated using the SUM function in Excel.
